# Optimizing ovine brucellosis serodiagnosis: evaluation of recombinant *Brucella* antigens and multi-antigen combinations for iELISA

**DOI:** 10.3389/fvets.2026.1775573

**Published:** 2026-03-09

**Authors:** Mixue Guo, Yan Huo, Tao Sun, Huqiang Dong, Jihui Yang, Yana Wang, Wei Zhao, Jiahui Song

**Affiliations:** 1 School of Basic Medical Sciences, Ningxia Medical University, Yinchuan, Ningxia, China; 2 Ningxia Key Laboratory of Prevention and Control of Common Infectious Diseases, Ningxia Medical University, Yinchuan, Ningxia, China; 3 School of Public Health, Ningxia Medical University, Yinchuan, Ningxia, China

**Keywords:** brucellosis, diagnostic performance, iELISA, multi-antigen cocktail, recombinant antigens

## Abstract

**Background:**

Brucellosis, a major zoonosis caused by *Brucella* spp., affects both animals and humans. Accurate serological diagnosis is crucial for effective disease control, yet current methods often lack optimal sensitivity and specificity. This study aimed to evaluate four recombinant *Brucella* antigens (rOmp19, rOmp2b, rOmp31, and rBP26) as diagnostic antigens, both individually and in combination, to identify an optimal multi-antigen cocktail for an improved indirect ELISA (iELISA).

**Methods:**

Four recombinant *Brucella* antigens were expressed in *E. coli* and purified by Ni-NTA affinity chromatography. A total of 224 sheep serum samples without a history of brucellosis vaccination were employed. The samples were classified as reference-positive (*n* = 20) or reference-negative (*n* = 204) based on parallel testing with a commercial cELISA kit and the serum agglutination test (SAT), following relevant WOAH recommendations. iELISA conditions were optimized by checkerboard titration. Diagnostic performance was assessed by calculating sensitivity, specificity, accuracy, and the area under the receiver operating characteristic curve (AUC).

**Results:**

Evaluation of single antigens revealed that rOmp19 had 100% sensitivity (20/20; 95% CI: 83.2%–100%) and the highest AUC (0.9931), but moderate specificity (93.62%; 95% CI: 89.1%–96.5%). Conversely, rOmp2b, rOmp31, and rBP26 each showed 100% specificity (95% CI: 98.2%–100%) with lower sensitivity (85%; 95% CI: 62.1%–96.8%) and AUCs (0.9547, 0.9498, and 0.9645). Multi-antigen formulations improved overall diagnostic balance. The two-antigen combination (rOmp19 + rBP26) increased specificity to 98.04% (95% CI: 95.0%–99.5%) while maintaining 90.0% sensitivity (95% CI: 68.3%–98.8%; AUC = 0.9841). The three-antigen cocktail (rOmp19 + rOmp31 + rBP26) achieved the optimal profile, retaining 100% sensitivity, elevating specificity to 99.51% (95% CI: 97.3%–99.9%), and yielding the best AUC among the multi-antigen combinations (AUC = 0.9850). Notably, adding a fourth antigen (rOmp19 + rOmp2b + rOmp31 + rBP26) substantially impaired performance, reducing sensitivity to 70%, specificity to 92.65%, and AUC to 0.8711.

**Conclusion:**

The three-antigen combination (rOmp19 + rOmp31 + rBP26) identified in this study achieves an optimal balance between sensitivity and specificity, supporting its potential to enhance the performance of brucellosis serodiagnosis. This work provides experimental evidence and constitutes an important step toward the development of multi-antigen iELISA-based brucellosis detection kits. However, further validation and optimization are required before clinical or field application.

## 1 Introduction

Brucellosis is a globally important zoonotic disease caused by infection with bacteria of the genus *Brucella*, with sustained impacts on livestock production and public health (1–3). In small ruminants, infection is most commonly associated with *Brucella melitensis*, and effective control depends heavily on timely flock-level surveillance and accurate serological diagnosis (4–6). According to the World Organization for Animal Health (WOAH) Terrestrial Manual (2024, Chapter 3.1.4), diagnosis and surveillance typically rely on a combination of bacteriological methods, molecular assays, and serological tests, selected according to purpose and available resources (7).

In China, brucellosis remains a priority disease in northern pastoral regions, where intensive husbandry practices increase the need for standardized screening (8, 9). Field practice commonly adopts agglutination-based screening, often using the Rose Bengal Plate Test (RBPT) followed by the SAT in serial testing algorithms, as recommended in national surveillance-related workflows; however, false positives and inconsistent performance across settings remain concerns (10–12). Consistent with this diagnostic demand, our epidemiological investigation in Ningxia Hui Autonomous Region (2021–2023) found a regional seropositivity rate of 21.13% among the surveyed population, underscoring the need for more reliable flock-level diagnostic tools to support early warning and source control at the animal–human interface (13).

Current diagnostic options each have practical limitations. Bacterial culture provides definitive confirmation but is slow and requires strict biosafety conditions (14); PCR is rapid and sensitive but can be constrained by cost, equipment requirements, and sample processing capacity, especially in resource-limited settings (15). Consequently, serology remains central for large-scale screening, but conventional assays using whole-cell or smooth lipopolysaccharide (S-LPS) antigens may suffer from cross-reactivity and a limited ability to address complex field scenarios (e.g., background antibodies, vaccination-related serological responses) (16–18). The WOAH manual summarizes these trade-offs and describes the broad use of indirect ELISA (iELISA) in small ruminants under appropriate standardization (19, 20).

To improve the specificity and standardization of iELISA, recent research has increasingly focused on recombinant protein antigens. Multiple studies have reported the diagnostic performance of iELISA based on single recombinant antigens, suggesting that antigen engineering can yield more controllable and standardized serological assays (21–23). For example, recombinant BP26 has been used for serological evaluation of *B. melitensis* infection in sheep, and recombinant Omp2b has shown high specificity and good sensitivity in animal serodiagnosis (22, 24). In addition, Omp19, an immunogenic outer membrane protein that can be recognized by the host immune system during infection, has also been investigated as a recombinant ELISA antigen and exhibits diagnostic potential (25). Meanwhile, recombinant Omp31 has been applied and compared in small ruminant populations (e.g., against RBPT), but its antibody responses may vary with host differences, infection stage, and epitope coverage, indicating that single-antigen formats may still show performance variability (26, 27).

Given the limited epitope coverage of single recombinant antigens and the heterogeneity of host antibody responses, recent studies have increasingly evaluated iELISAs using mixed recombinant antigens as coating antigens for brucellosis serodiagnosis (28–30). By combining antigens with complementary immunological properties in a single assay, mixed-antigen formats may broaden the antibody-recognizable epitope repertoire and improve robustness across heterogeneous field sera (31–33). For example, an iELISA using a mixed coating of rOmp19, rOmp25, and rOmp31 has been reported to improve overall diagnostic performance in animal sera. Other combinations, such as rOmp25/rOmp31/rBP26 and rOmp10/rOmp19/rOmp28, have also shown promising diagnostic performance (28, 33, 34).

Accordingly, this study expressed and purified four recombinant *Brucella* antigens—Omp19, Omp2b, Omp31, and BP26—and systematically evaluated their ability to detect *Brucella*-specific IgG in sheep sera using iELISA. By comparing single antigens with different antigen combinations in terms of sensitivity, specificity, and overall agreement, we aimed to identify an optimized antigen strategy that can support more accurate serological detection of sheep brucellosis.

## 2 Materials and methods

### 2.1 Expression and purification of recombinant proteins

The coding sequences of *Brucella* outer membrane proteins *omp19* (GenBank: ON813304.1), *omp2b* (AM712080.1) and *omp31* (MK611562.1) were retrieved from GenBank, PCR-amplified, and cloned into pET-30a (+). Recombinant plasmids were confirmed by *EcoRI/HindIII* digestion and Sanger sequencing and subsequently transformed into *Escherichia coli* BL21 (DE3). A BL21 (DE3) glycerol stock harboring BP26 in pET-30a (+), previously constructed in our laboratory, was used as described (35). Transformants were cultured in LB containing kanamycin (100 μg/mL; Biotopped, Beijing) to an OD_600_ of approximately 0.6, followed by induction with IPTG (Solarbio, Beijing). Because recombinant proteins differ in expression kinetics and solubility, induction parameters were screened at small scale, and conditions were selected based on target yield, soluble fraction, and band quality on SDS-PAGE. The final induction conditions were: rOmp19 and rOmp31 (0.5 mM IPTG, 16 °C, 16 h), rOmp2b (0.2 mM IPTG, 37 °C, 14 h), and rBP26 (0.5 mM IPTG, 29 °C, 8 h; optimized previously in our laboratory).

For predominantly soluble proteins (rOmp19 and rBP26), cell pellets were washed with PBS and resuspended in 1 × binding buffer (140 mM NaCl, 2.7 mM KCl, 10 mM Na2HPO4, 1.8 mM KH2PO4, pH 7.3) supplemented with lysozyme (1 mg/mL) and PMSF (1 mmol/L). After incubation on ice for 30 min, cells were disrupted by sonication (300 W, 1.5 s on/1.0 s off; total 5 min; five cycles). Lysates were clarified by centrifugation (4 °C, 12,000 rpm, 30 min), and the supernatants were purified at 4 °C using a His·Bind Purification Kit (Merck, Germany). For inclusion-body proteins (rOmp2b and rOmp31), cell pellets were resuspended in denaturing lysis buffer containing 8 M urea and incubated at 4 °C with agitation overnight. After clarification (4 °C, 12,000 rpm, 30 min), supernatants were subjected to refolding by stepwise dialysis at 4 °C using a 10-kDa MWCO dialysis membrane. The refolding buffer contained 50 mM Tris-Cl, 50 mM L-arginine, 5 mM EDTA, 50 mM NaCl, 5% glycerol, and 0.5% Triton X-100, with urea concentrations reduced sequentially (6, 4, 2, 1, and 0 M). Each step was performed for 12 h with buffer replacement every 4 h, using a buffer volume approximately 200-fold greater than the sample volume. Proteins were then dialyzed against PBS, filtered through a 0.45 μm membrane, and purified at 4 °C by Ni-NTA affinity chromatography. Purified proteins were stored at 4 °C for short-term use (≤1 month) or at −80 °C for long-term storage.

### 2.2 Quality control of recombinant proteins: purity, concentration, and endotoxin level

Purified recombinant proteins were analyzed by SDS-PAGE followed by Coomassie Brilliant Blue staining to assess purity, and protein concentrations were determined using the Bradford assay (Beyotime, Shanghai). To minimize endotoxin interference in downstream immunological assays, all proteins were processed with the ToxinEraser™ Endotoxin Removal Kit (GenScript, Nanjing). The residual endotoxin level of each protein preparation was quantified using a Limulus amebocyte lysate (LAL) assay and was < 0.1 EU/mL.

### 2.3 Establishment and condition optimization of iELISA

An iELISA was established and optimized by checkerboard titration using commercially available standard *Brucella*-positive and-negative reference sera. The reference sera were purchased from Harbin Pharmaceutical Group Bio-vaccine Co., Ltd. (China), including a standard positive serum (Veterinary Drug Approval No. 080078011; lot 202,101) and a standard negative serum (Veterinary Drug Approval No. (2016) 080078011; lot 202,001). Coating antigens included four individual recombinant proteins (rOmp19, rOmp2b, rOmp31, and rBP26) and three antigen combinations (rOmp19 + rBP26; rOmp19 + rOmp31 + rBP26; and rOmp19 + rOmp2b + rOmp31 + rBP26). Antigens were diluted in carbonate–bicarbonate coating buffer (35 mM NaHCO₃ and 15 mM Na₂CO₃; pH 9.5). For mixed-antigen formats, component antigens were prepared at equal mass concentrations and combined accordingly; the optimal total coating concentration for each single-antigen or mixed-antigen preparation was subsequently determined by checkerboard titration.

Optimization parameters included: (i) coating concentration of the single antigen or antigen mixture, (ii) serum dilution, and (iii) working dilution of the HRP-conjugated secondary antibody (Rabbit anti-sheep IgG (H/L): HRP; BIO-RAD, 5184–2,504). The condition that maximized the OD_450_ ratio of the standard positive serum to the standard negative serum (P/N) was selected. Unless otherwise specified, plates were coated overnight at 4 °C, washed with PBST (1 × PBS containing 0.05% Tween 20; five washes, 3 min each), and blocked with 5% skim milk in PBST at 37 °C for 2 h. Diluted sera were then incubated at 37 °C for 2 h, followed by incubation with the optimized dilution of the secondary antibody at 37 °C for 1 h. Color development was performed using TMB substrate (Solarbio, PR1210-2) in the dark for 15 min and terminated with stop solution (Solarbio, C1058). Absorbance was measured at 450 nm. All assays were performed in triplicate wells.

### 2.4 Sheep serum sampling and screening

A total of 284 sheep blood samples were collected at a large slaughterhouse in Yinchuan, Ningxia, China. Samples were randomly selected from the slaughter line on the sampling days. According to slaughterhouse personnel, most sheep processed during the sampling period originated from different small-scale free-range households in the surrounding areas and were approximately 6–8 months of age. None of the sampled sheep had been vaccinated against brucellosis; however, the stage of infection and the presence of potential co-infections could not be determined. Whole blood was allowed to clot at room temperature for 2 h and was then centrifuged at 4,000 rpm for 15 min. Serum was separated, aliquoted, and stored at −80 °C until further testing.

All sera were screened for anti-*Brucella* antibodies using two serological assays: a commercial competitive ELISA (cELISA; Shenzhen Kangbide Biotechnology Co., Ltd.; kit No. 261411–2,401) and the SAT. The cELISA was performed according to the manufacturer’s instructions; inhibition percentage (PI) was calculated as PI = (OD_NC_ − OD_sample_)/OD_NC_ × 100%, and PI ≥ 50% was interpreted as positive. SAT was performed using an antigen preparation primarily based on the *Brucella* S2 strain (CVCC70502) following the product instructions; agglutination of “++” or higher at a 1:50 serum dilution was interpreted as positive.

For subsequent iELISA performance evaluation, a stringent composite reference definition was applied to reduce misclassification: samples concordantly positive by both assays (cELISA+/SAT+) were defined as candidate reference-positive, and samples concordantly negative (cELISA−/SAT−) were defined as reference-negative; discordant samples were excluded from diagnostic performance analyses. To further increase confidence in reference positivity, all concordantly positive sera were re-tested using a commercial colloidal gold lateral flow immunoassay (LFIA) (Shenzhen Kerunda Bioengineering Co., Ltd.; Cat. No. KRD-BR-20) and interpreted according to the manufacturer’s instructions (read at 10–15 min; both control and test lines visible = positive). Only LFIA-confirmed concordantly positive sera were included as high-confidence seropositive reference samples.

### 2.5 Serological evaluation and statistical analysis of recombinant-antigen iELISAs

iELISA testing was performed on 224 sheep serum samples under the optimized conditions. For each antigen (single or mixed coating), OD_450_ values were used to construct receiver operating characteristic (ROC) curves in GraphPad Prism v10.1.2 (GraphPad Software, United States), and the AUC was calculated. Reference-positive and reference-negative status for ROC analysis was defined *a priori* using the composite serological reference described in Section 2.4: sera classified as cELISA+/SAT+/LFIA+ were treated as reference-positive, whereas sera classified as cELISA−/SAT − were treated as reference-negative. The optimal cutoff was determined using the Youden index (J = sensitivity + specificity − 1). Based on the selected cutoff, samples were classified as true positives, true negatives, false positives, and false negatives, and diagnostic indices including, sensitivity, specificity, accuracy, positive predictive value (PPV), negative predictive value (NPV), false-positive rate (FPR), and false-negative rate (FNR) were calculated. Exact (Clopper–Pearson) 95% confidence intervals (CIs) were computed for sensitivity, specificity, PPV, and NPV; 95% CIs for FPR and FNR were derived from the corresponding proportions (FPR = 1 − specificity; FNR = 1 − sensitivity) using the same exact binomial method. For comparisons among coating formats (single antigens vs. antigen combinations), group differences were assessed by one-way ANOVA followed by Tukey’s multiple-comparisons *post hoc* test. A *p* value < 0.05 was considered statistically significant.

## 3 Results

### 3.1 Plasmid construction and production of recombinant proteins

**Figure 1:**
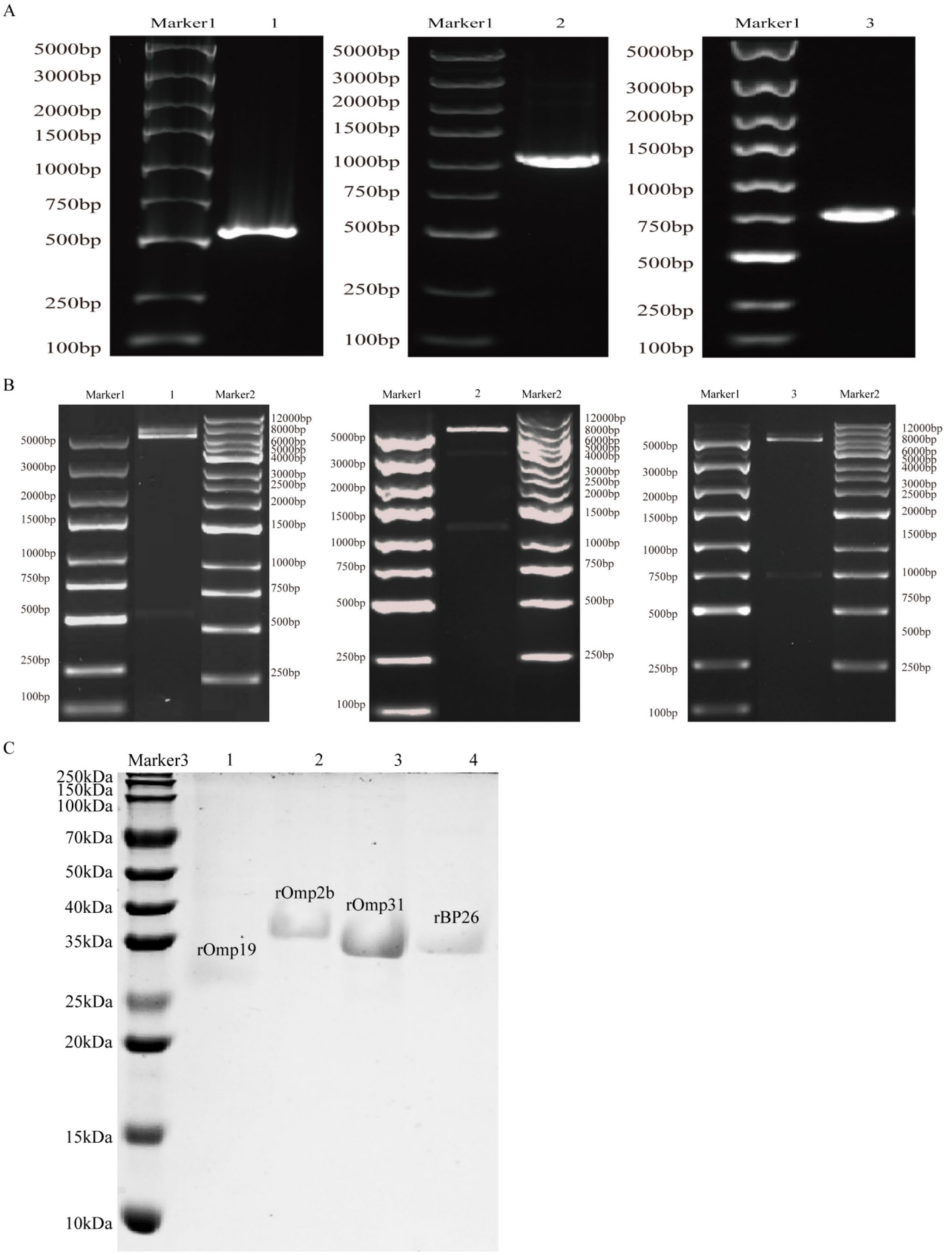
Construction of recombinant plasmids and SDS-PAGE analysis of purified recombinant proteins. **(A)** PCR amplification of *omp19*, *omp2b*, and *omp31*. Marker 1, DNA ladder (100–5,000 bp). Lanes 1–3, PCR products of *omp19* (534 bp), *omp2b* (1,089 bp), and *omp31* (723 bp), respectively. **(B)** *EcoRI/HindIII* restriction analysis of pET-30a/Omp19, pET-30a/Omp2b, and pET-30a/Omp31. Marker 2, DNA ladder (250–12,000 bp). Lanes 1–3, digested plasmids showing released inserts of the expected lengths. **(C)** Coomassie-stained SDS-PAGE of purified recombinant proteins. Marker 3, protein molecular weight marker (10–250 kDa). Lanes 1–4, rOmp19, rOmp2b, rOmp31, and rBP26, respectively.

PCR amplification generated *omp19*, *omp2b*, and *omp31* amplicons of the expected sizes (534 bp, 1,089 bp, and 723 bp, respectively) (Figure 1A). The corresponding recombinant plasmids (pET-30a/Omp19, pET-30a/Omp2b, and pET-30a/Omp31) were further verified by EcoRI/HindIII restriction digestion, which released insert fragments consistent with the expected lengths (Figure 1B). In addition, Sanger sequencing confirmed the correct insert sequences and vector–insert junctions, and the results are provided in Supplementary Figure S1.

Following IPTG induction and affinity purification, Coomassie-stained SDS-PAGE showed a single major band at approximately 27 kDa (rOmp19), 37 kDa (rOmp2b), 35 kDa (rOmp31), and 36 kDa (rBP26), in agreement with their predicted molecular weights (Figure 1C). To support an objective assessment of protein purity, Coomassie-stained gels were quantified by densitometry in ImageJ. Apparent purity was calculated as the background-subtracted integrated density of the target band divided by the total lane signal; all four recombinant proteins showed apparent purities of ≥95% (see Supplementary Table S1 for densitometry outputs). Protein concentrations determined by the Bradford assay were 2.30 mg/mL (rOmp19), 1.10 mg/mL (rOmp2b), 1.23 mg/mL (rOmp31), and 1.35 mg/mL (rBP26) (Figure 1).

### 3.2 Screening of serum samples

**Figure 2:**
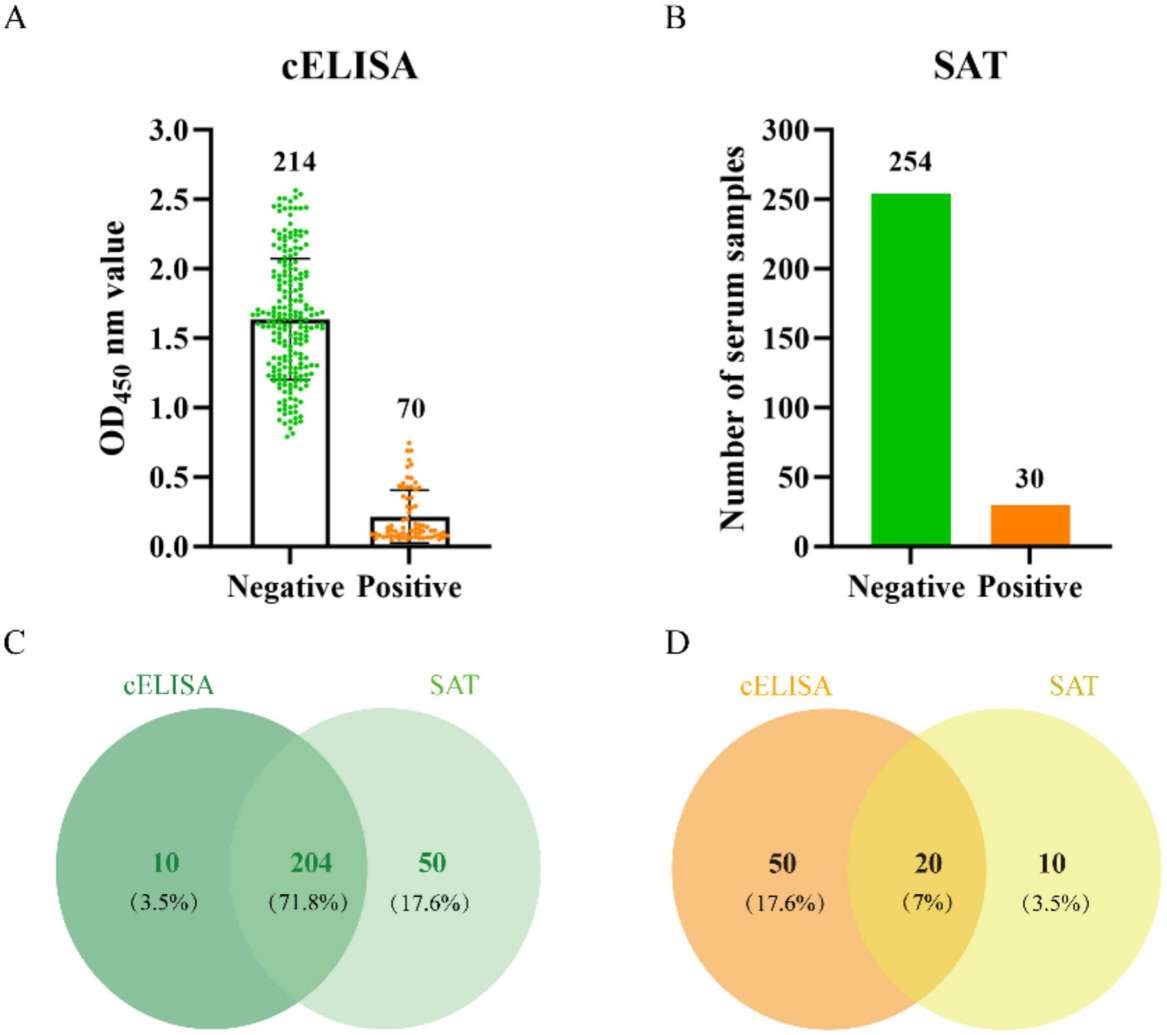
Parallel serological screening of sheep sera by cELISA and SAT and concordance between assays. **(A)** cELISA results for the screened sera. **(B)** SAT results for the same sera. **(C,D)** Venn diagrams showing concordance between cELISA and SAT for negative **(C)** and positive **(D)** classifications; overlaps indicate concordant results, whereas non-overlapping areas indicate discordant results. Values are shown as *n* (%), calculated using the total number of sera (*n* = 284) as the denominator.

To screen sheep sera for anti-*Brucella* antibodies, 284 samples were tested in parallel using a commercial competitive ELISA (cELISA) and the serum tube agglutination test (SAT). cELISA identified 70/284 samples as positive and 214/284 as negative (Figure 2A), whereas SAT identified 30/284 as positive and 254/284 as negative (Figure 2B). Concordance between the two assays was incomplete: 204/284 sera were concordantly negative (cELISA−/SAT−; Figure 2C) and 20/284 were concordantly positive (cELISA+/SAT+; Figure 2D). The remaining 60/284 samples were discordant, including 50 cELISA+/SAT − and 10 cELISA−/SAT+. Accordingly, for subsequent evaluation of recombinant-antigen iELISAs, we defined a composite serological reference standard to reduce misclassification: concordantly negative sera (*n* = 204) were designated as the reference-negative panel and concordantly positive sera (*n* = 20) as the candidate reference-positive panel, whereas discordant sera (*n* = 60) were excluded from performance analyses. To increase confidence in the candidate reference-positive panel, all 20 concordantly positive sera (cELISA+/SAT+) were further re-tested using a commercial colloidal gold LFIA, and all 20 were confirmed positive (Supplementary Figure S2) (Figure 2).

### 3.3 Optimization and establishment of single-antigen iELISA conditions

**Figure 3:**
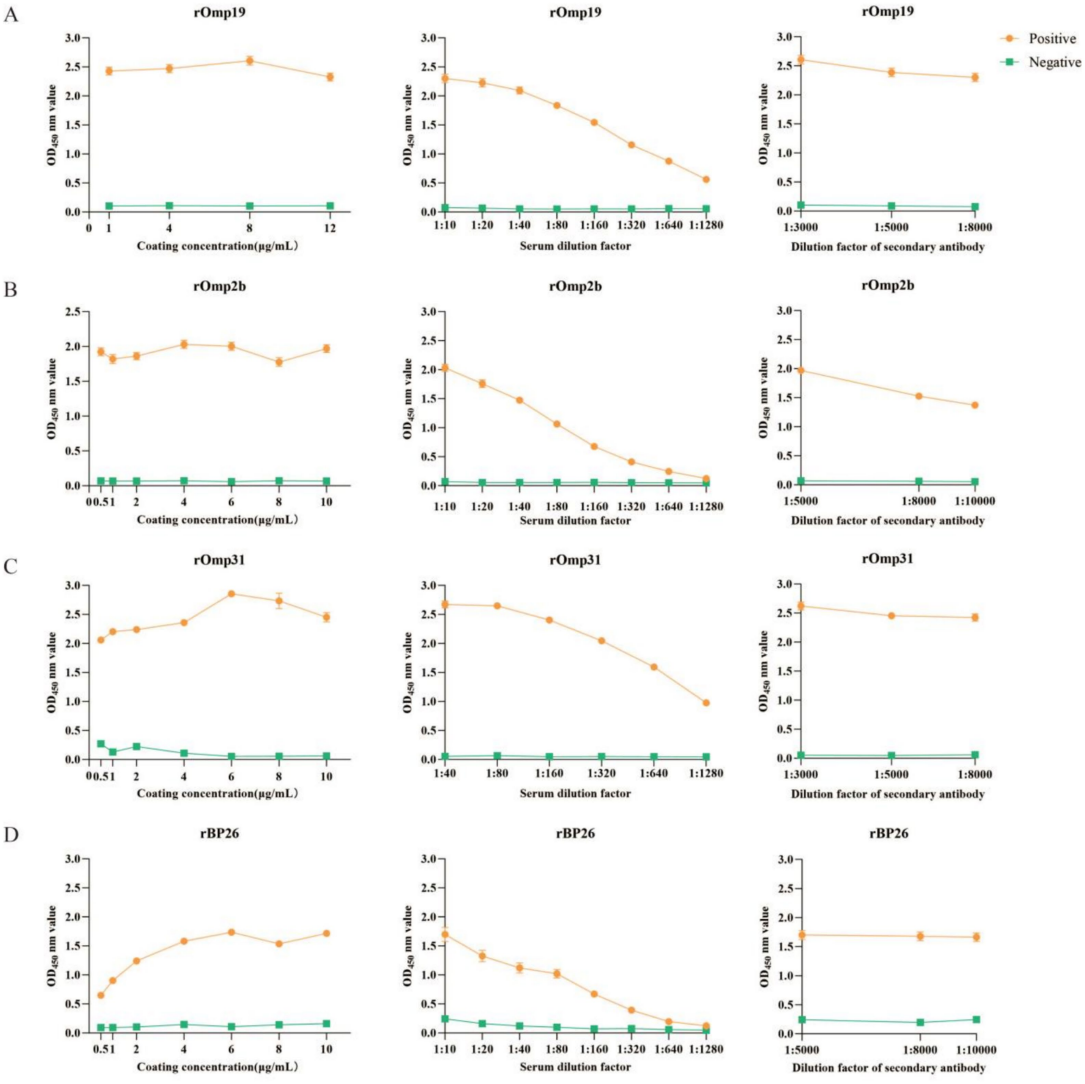
Optimization of single-antigen iELISA conditions for recombinant Brucella antigens by checkerboard titration. For each antigen, coating concentration (left), serum dilution (middle), and HRP-conjugated secondary antibody dilution (right) were optimized using standard Brucella-positive and -negative reference sera. Data points represent mean OD450 ± SD (technical triplicates). Working conditions were selected based on the maximum P/N ratio. **(A)** rOmp19; **(B)** rOmp2b; **(C)** rOmp31; **(D)** rBP26.

Before evaluating diagnostic performance using the screened sera, iELISA conditions were optimized for each single recombinant coating antigen by checkerboard titration (Figures 3A–D). The final working conditions for the single-antigen formats were: rOmp19, a coating concentration of 8 μg/mL with a serum dilution of 1:10 and a secondary antibody dilution of 1:3000 (Figure 3A); rOmp2b, 10 μg/mL with 1:10 and 1:5000 (Figure 3B); rOmp31, 6 μg/mL with 1:40 and 1:3000 (Figure 3C); and rBP26, 10 μg/mL with 1:10 and 1:5000 (Figure 3D). These optimized settings were used in subsequent iELISA testing of the screened sheep sera (*n* = 224) (Figure 3).

For each antigen, the coating concentration (left), serum dilution (middle), and HRP-conjugated secondary antibody dilution (right) were optimized using commercially available *Brucella* standard positive and negative reference serum. Data points represent mean OD_450_ ± SD from technical triplicates (three wells) for the positive (orange circles) and negative (green squares) sera. The working conditions for each antigen were selected based on the maximum P/N ratio, calculated as mean OD_450_ (positive) divided by mean OD_450_ (negative) under the same condition. (A) rOmp19; (B) rOmp2b; (C) rOmp31; (D) rBP26.

### 3.4 Evaluation of diagnostic performance of iELISA for single recombinant antigens

**Figure 4:**
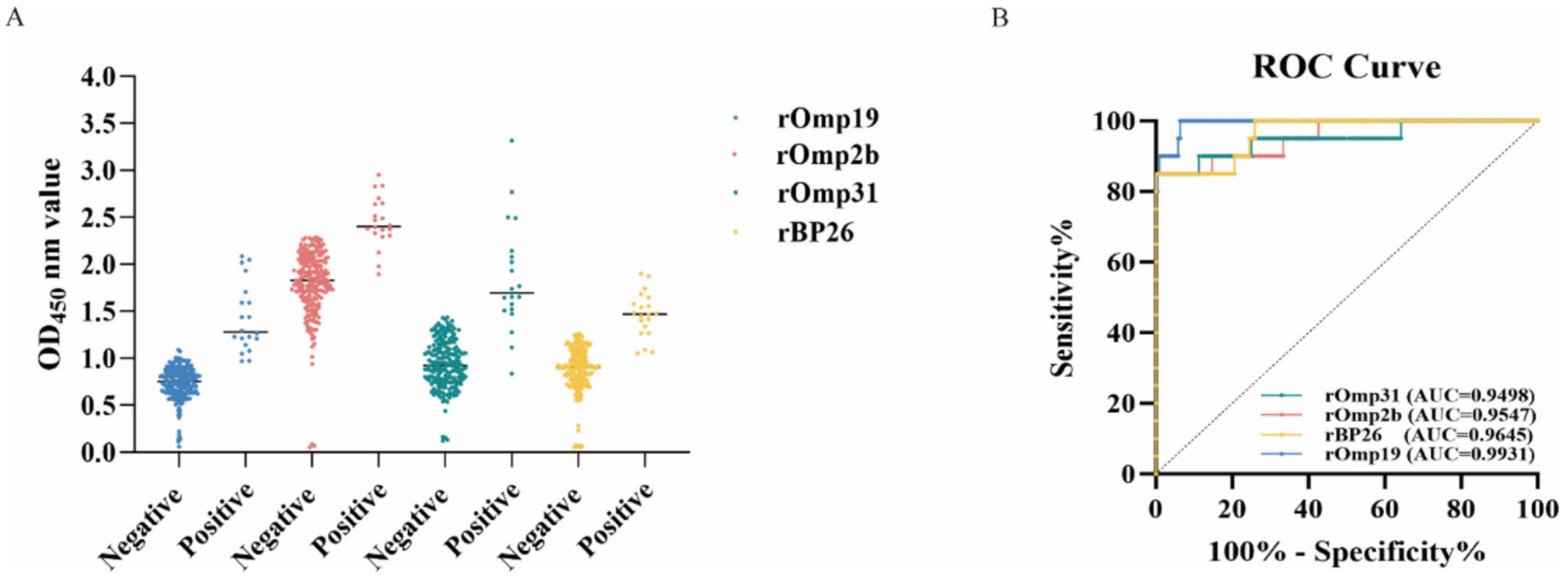
Diagnostic performance of single-antigen recombinant iELISAs. **(A)** Scatter plots of OD_450_ values for sera tested with iELISAs coated with rOmp19, rOmp2b, rOmp31, or rBP26; horizontal lines indicate the median. **(B)** ROC curves for each antigen, with the corresponding AUC values shown.

Using the screened sheep serum (*n* = 224; 20 reference-positive and 204 reference-negative), iELISAs based on single recombinant antigens (rOmp19, rOmp2b, rOmp31, or rBP26) showed clear separation between groups, with higher OD_450_ values in reference-positive sera across all four assays (Figure 4A). ROC analysis further demonstrated good discriminatory performance for each antigen (Figure 4B). Evaluation of single antigens revealed that rOmp19 achieved 100% sensitivity (20/20; 95% CI: 83.2%–100%) and the highest AUC (0.9931; 95% CI: 0.9840–1.0000), but showed comparatively lower specificity (93.62%; 191/204; 95% CI: 89.1%–96.5%). In contrast, rBP26 exhibited a sensitivity of 85.0% (17/20; 95% CI: 62.1%–96.8%) and a specificity of 100% (204/204; 95% CI: 98.2%–100%), with an AUC of 0.9645 (95% CI: 0.9260–1.0000). Similarly, rOmp2b showed a sensitivity of 85.0% (17/20; 95% CI: 62.1%–96.8%) and a specificity of 100% (204/204; 95% CI: 98.2%–100%), with an AUC of 0.9547 (95% CI: 0.9026–1.0000), while rOmp31 yielded the same sensitivity (85.0%; 17/20; 95% CI: 62.1%–96.8%) and specificity (100%; 204/204; 95% CI: 98.2%–100%), with an AUC of 0.9498 (95% CI: 0.8845–1.0000) (Figure 4).

At the Youden index–derived cutoffs, rOmp19 prioritized sensitivity at the expense of a modest reduction in specificity, whereas rOmp2b, rOmp31, and rBP26 favored higher specificity with a corresponding decrease in sensitivity. Detailed diagnostic indices are summarized in Table 1.

**Table 1:** Diagnostic performance of single-antigen recombinant iELISAs for detecting *Brucell*a-specific antibodies in sheep sera.

**Antigen**	**Positive**		**Negative**		**Cut-Off value**	**Sensitivity (%)**	**Specificity (%)**	**Accuracy (%)**	**PPV (%)**	**NPV (%)**	**FPR (%)**	**FNR (%)**
	TP	FP	TN	FN								
rOmp19	20	13	191	0	0.9655	100.00	93.62	94.20	60.61	100.00	6.38	0.00
rOmp2b	17	0	204	3	2.289	85.00	100.00	98.66	100.00	98.55	0.00	15.00
rOmp31	17	0	204	3	1.452	85.00	100.00	98.66	100.00	98.55	0.00	15.00
rBP26	17	0	204	3	1.263	85.00	100.00	98.66	100.00	98.55	0.00	15.00

Diagnostic indices were calculated using 224 sera (20 reference-positive and 204 reference-negative). Cut-off values were determined by ROC analysis using the Youden index.

### 3.5 Optimization of iELISA conditions for mixed-antigen combinations

Building on the optimized single-antigen iELISAs, three mixed-antigen coating formats were further evaluated (rOmp19 + rBP26, rOmp19 + rOmp31 + rBP26, and rOmp19 + rOmp2b + rOmp31 + rBP26). For each combination, the working conditions were selected by checkerboard titration to maximize the P/N ratio obtained with the commercial standard *Brucella*-positive and -negative sera.

**Figure 5:**
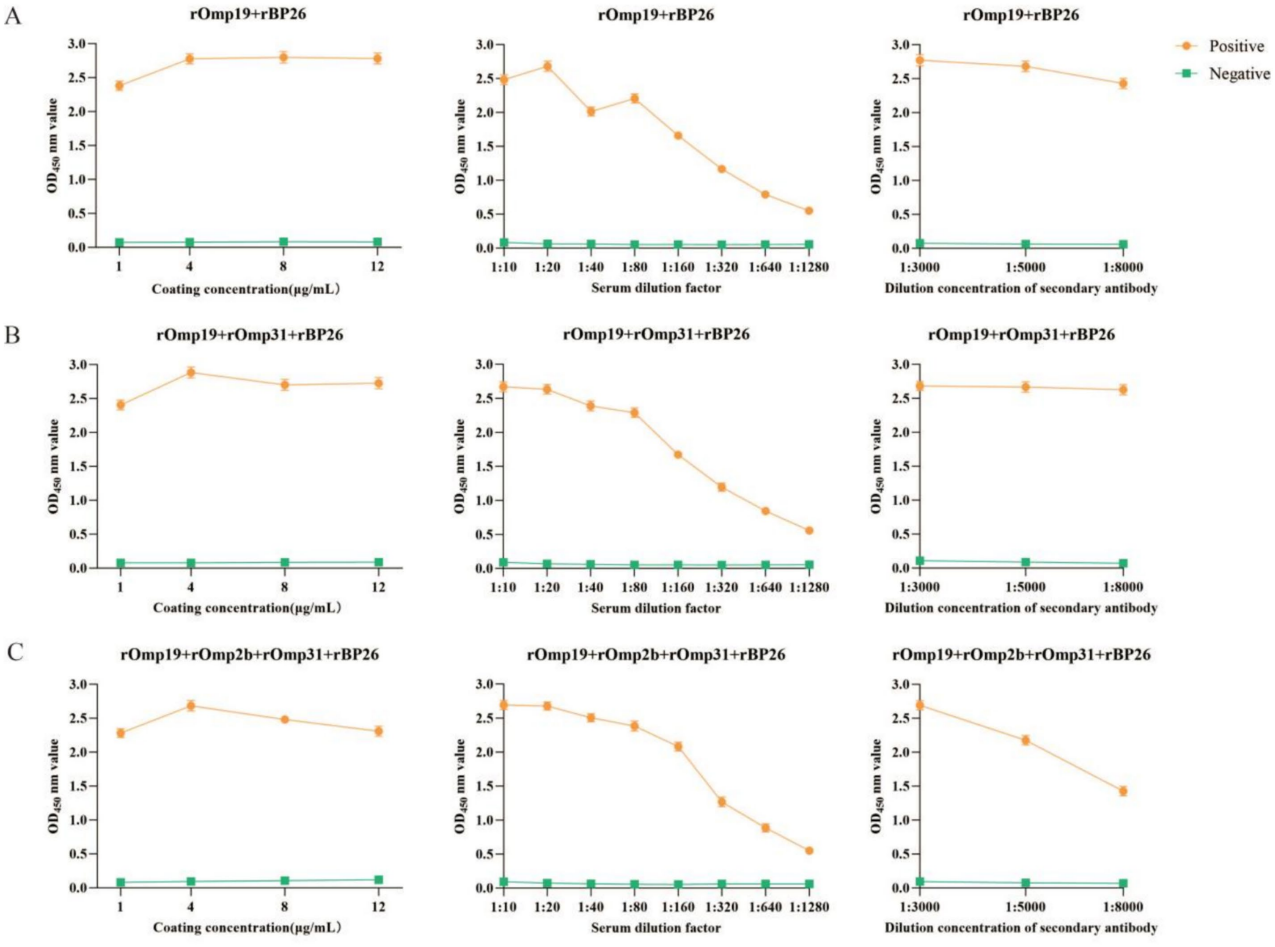
Checkerboard titration for optimizing mixed-antigen iELISAs. Coating concentration (left), serum dilution (middle), and secondary antibody dilution (right) were optimized using standard Brucella-positive and -negative reference sera. Working conditions were selected based on the maximum P/N ratio. **(A)** rOmp19 + rBP26; **(B)** rOmp19 + rOmp31 + rBP26; **(C)** rOmp19 + rOmp2b + rOmp31 + rBP26.

As shown in Figure 5, the optimal conditions for the dual-antigen format (rOmp19 + rBP26) were a total coating concentration of 8 μg/mL with serum dilution 1:20 and secondary antibody dilution 1:3000 (Figure 5A). For the triple-antigen format (rOmp19 + rOmp31 + rBP26), the optimal settings were 4 μg/mL with serum dilution 1:10 and secondary antibody dilution 1:5000 (Figure 5B). The four-antigen format (rOmp19 + rOmp2b + rOmp31 + rBP26) achieved the highest P/N under a total coating concentration of 4 μg/mL, serum dilution 1:10, and secondary antibody dilution 1:3000 (Figure 5C). Under these selected conditions, all mixed-antigen formats produced clear separation between the positive and negative standards with low background signals (Figure 5).

Coating concentration, serum dilution, and secondary antibody dilution were optimized for each mixed-antigen format using commercial standard *Brucella*-positive and -negative sera, with the working condition selected by the maximum P/N ratio. (A) rOmp19 + rBP26; (B) rOmp19 + rOmp31 + rBP26; (C) rOmp19 + rOmp2b + rOmp31 + rBP26.

### 3.6 Evaluation of diagnostic performance of combination antigens

**Figure 6:**
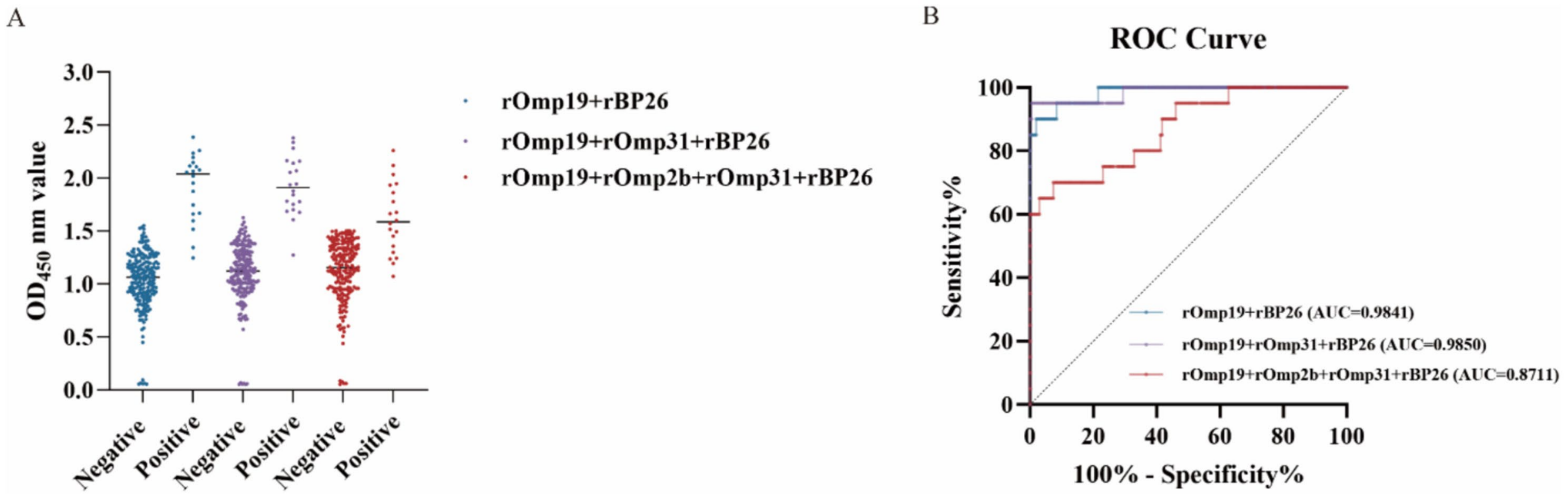
Diagnostic performance of mixed-antigen iELISAs. **(A)** OD_450_ distributions obtained with each mixed-antigen coating format for sera with reference status defined in the methods. Horizontal bars indicate the median. **(B)** ROC curves for each mixed-antigen iELISA. AUCs (with 95% CIs) were calculated as described in the methods.

Using the same reference serum panel defined in Section 2.4, we evaluated three mixed-antigen coating formats (rOmp19 + rBP26; rOmp19 + rOmp31 + rBP26; and rOmp19 + rOmp2b + rOmp31 + rBP26) under their respective optimized conditions. In all three formats, OD_450_ values showed separation between reference-positive and reference-negative sera (Figure 6A). ROC analysis indicated good discrimination for the rOmp19 + rOmp31 + rBP26 and rOmp19 + rBP26 formats, whereas the four-antigen mixture (rOmp19 + rOmp2b + rOmp31 + rBP26) showed comparatively lower discrimination (Figure 6B).

For transparency and reproducibility, the ROC-derived cutoffs (OD_450_) for each antigen combination—selected by maximizing the Youden index—are provided in Table 2 together with the corresponding confusion-matrix–based diagnostic indices. Within this reference panel and under the current assay conditions, the rOmp19 + rOmp31 + rBP26 combination showed a comparatively favorable sensitivity–specificity trade-off among the evaluated mixed-antigen formats. The rOmp19 + rBP26 combination tended to improve specificity relative to rOmp19 alone, although some false-positive classifications were still observed. By contrast, inclusion of rOmp2b (rOmp19 + rOmp2b + rOmp31 + rBP26) was associated with reduced overall performance in this dataset, consistent with an increased false-negative burden (Table 2). Importantly, these findings are presented to contextualize the behavior of different antigen mixtures and to prioritize candidate combinations for further optimization and external validation, rather than to support immediate application for high-precision serological diagnosis.

**Table 2:** Comparison of diagnostic performance of iELISAs using different combinations of recombinant antigens for detecting *Brucella*-specific antibodies in sheep sera.

**Antigen**	**Positive**		**Negative**		**Cut-Off value**	**Sensitivity (%)**	**Specificity (%)**	**Accuracy (%)**	**PPV (%)**	**NPV (%)**	**FPR (%)**	**FNR (%)**
	TP	FP	TN	FN								
rOmp19 + rBP26	18	4	200	2	1.498	90.00	98.04	97.32	81.82	99.01	1.96	10.00
rOmp19 + rOmp31 + rBP26	20	1	203	0	1.595	100.00	99.51	99.55	95.24	100.00	0.49	0.00
rOmp19 + rOmp2b + rOmp31 + rBP26	14	15	189	6	1.448	70.00	92.65	90.63	48.28	96.92	7.35	30.00

Indices and cut-offs were calculated as in Table 1, using the same reference serum and ROC-based cut-off selection.

### 3.7 Performance of different iELISA antigen systems in samples with discordant cELISA and SAT

To explore the behavior of iELISA under diagnostically uncertain conditions, a total of 60 serologically discordant serum samples, defined by inconsistent results between cELISA and SAT, were subjected to further analysis rather than being excluded. These samples included 50 cELISA-positive/SAT-negative (cELISA+/SAT-) sera and 10 cELISA-negative/SAT-positive (cELISA-/SAT+) sera. Indirect ELISA was performed using four single-antigen systems (rOmp19, rOmp2b, rOmp31, and rBP26) and three multi-antigen combinations (rOmp19 + rBP26, rOmp19 + rOmp31 + rBP26, and rOmp19 + rOmp2b + rOmp31 + rBP26). OD_450_ values were interpreted according to the predefined cutoff values established for each antigen or antigen combination (rOmp19, 0.9655; rOmp2b, 2.289; rOmp31, 1.452; rBP26, 1.263; rOmp19 + rBP26, 1.498; rOmp19 + rOmp31 + rBP26, 1.595; rOmp19 + rOmp2b + rOmp31 + rBP26, 1.448), and the results were compared in a descriptive manner (Supplementary Figure S9) (Supplementary Table S2).

Among the cELISA+/SAT discordant samples (*n* = 50), substantial variation in iELISA classification was observed across different antigen systems. The rOmp19 single-antigen iELISA classified 38/50 samples (76.0%) as positive, representing the highest positive proportion among single-antigen formats, followed by the four-antigen combination, which classified 36/50 samples (72.0%) as positive. In contrast, rOmp2b showed the lowest positive classification rate (20/50, 40.0%), while rOmp31 and rBP26 classified 23/50 (46.0%) and 28/50 (56.0%) samples as positive, respectively. Among the antigen combinations, rOmp19 + rBP26 and the three-antigen panel (rOmp19 + rOmp31 + rBP26) classified 60.0% (30/50) and 54.0% (27/50) of samples as positive, respectively. In the cELISA-/SAT + discordant group (n = 10), a similarly antigen-dependent pattern was observed. rOmp19, rOmp2b, rBP26, and the four-antigen combination each classified 8/10 samples (80.0%) as positive. The rOmp19 + rBP26 combination classified 7/10 samples (70.0%) as positive, whereas rOmp31 and rOmp19 + rOmp31 + rBP26 each classified 6/10 samples (60.0%) as positive. The analysis shows that iELISA results in samples with discordant cELISA and SAT outcomes vary substantially depending on the antigen system used. Different single-antigen and multi-antigen formats classified the same discordant sera differently.

## 4 Discussion

This study established an iELISA using four *Brucella* recombinant outer membrane–associated proteins (rOmp19, rOmp2b, rOmp31, and rBP26) and evaluated single and multi-antigen formats with sera from naturally infected sheep. Overall, rOmp19 showed stronger detection capability as a single antigen but with relatively constrained specificity (93.62%), whereas rOmp2b, rOmp31, and rBP26 tended to provide higher specificity (100.00%) with moderate sensitivity (85.00%). Under the current assay conditions and within this dataset, the tri-antigen panel (rOmp19 + rOmp31 + rBP26) showed a comparatively favorable sensitivity–specificity profile (100 and 99.51%, respectively), whereas adding rOmp2b to generate a four-antigen mixture did not result in an observable improvement and was associated with reduced overall performance. These findings suggest that multi-antigen diagnostics depend more on rational complementarity and parameter matching than on simply increasing antigen numbers.

In the context of prior work, serological assays targeting LPS often achieve high sensitivity but can be affected by cross-reactivity, whereas protein-based assays may improve specificity but may miss some early-stage infections or low-titer sera (1). Omp family proteins and BP26 have been repeatedly investigated as diagnostic antigens, yet reported performance varies across host species, infection stage, and sample sets. A key factor influencing the immune response to these antigens is the stage of infection. During early infections, the immune system may not have developed a strong antibody response, resulting in lower antibody titers that could reduce the sensitivity of protein-based assays, while later stages may show higher titers. Importantly, as described in the Methods, all sampled sheep in this study were not vaccinated against brucellosis; therefore, the present findings reflect antibody responses in an unvaccinated, naturally exposed population and cannot be extrapolated to vaccinated herds or DIVA-related applications. Consistent with the general “complementary antigen” rationale, our data indicate that rOmp19 may function as a sensitivity-oriented component, while rOmp31 and rBP26 contribute more to specificity control (36). The improved performance of the tri-antigen panel aligns with multi-antigen strategies aimed at broadening epitope coverage and reducing stage-dependent false negatives. Conversely, the lack of improvement in the four-antigen format is compatible with reports that increasing antigen complexity may elevate background and destabilize cutoffs.

The observed differences among antigens likely reflect a combination of antigen localization, epitope exposure, and antibody kinetics (37). As a lipoprotein, rOmp19 may elicit earlier or broader humoral responses, which could explain higher sensitivity, while also potentially increasing non-specific binding (38). rOmp31 may provide more specific epitopes but could display stage-dependent antibody dynamics. BP26, a periplasmic protein, may have limited epitope accessibility in intact bacteria and thus may be less sensitive in early infection. The reduced performance of the four-antigen mixture may point to coating-related interference in mixed systems, such as limited epitope presentation due to high coating density (epitope masking/conformational changes), accumulated background reducing signal-to-noise ratio, and competition for adsorption sites causing steric effects (39). Future studies should explore the impact of infection stage on these phenomena, as understanding these interactions could lead to improved diagnostic strategies. For rOmp2b in particular, its adsorption behavior or conformational stability on solid supports could differ from the other antigens, potentially increasing variability in mixed coatings; however, this remains a hypothesis that requires targeted verification.

Importantly, to address diagnostic uncertainty present in real-world screening, we further examined sera with discordant reference results (cELISA vs. SAT) rather than excluding them. In these serologically discordant samples, iELISA classifications were strongly antigen-dependent: rOmp19 tended to classify a higher proportion of discordant sera as positive in both discordant subsets, whereas rOmp2b and rOmp31 showed lower positive classification rates in the cELISA⁺/SAT^−^ group; multi-antigen mixtures also produced non-uniform reclassification patterns (Supplementary Figure S9; Supplementary Table S2). Notably, increasing antigen number did not lead to a monotonic increase in positive classifications, and some sera switched classification across different mixtures, suggesting that mixed-antigen coatings can introduce competing effects (e.g., altered adsorption efficiency, epitope masking, and background accumulation) that interact with cutoff definition. These observations do not imply that iELISA “resolves” discordance between cELISA and SAT; rather, they highlight the heterogeneous antibody landscapes in ambiguous samples and underscore the need for cautious interpretation and further validation in well-characterized cohorts. From a development perspective, discordant sera provide an informative stress-test set for refining antigen ratios, coating strategies (mixed vs. sequential), and cutoff robustness before broader field deployment is considered.

## 5 Limitations

Several limitations should be noted. First, sera were collected at a slaughterhouse and lacked individual metadata, which may introduce heterogeneity and complicate stage-related interpretation. Second, bacteriological culture was not feasible; therefore, a composite reference (cELISA, SAT, and a colloidal gold assay) was used. While practical, this approach may still involve imperfect reference standard bias (40). Third, the animals originated from dispersed smallholder farms, and the apparent brucellosis positivity rate in this source population was relatively low; consequently, the number of reference-positive sera was limited, which may increase uncertainty in sensitivity estimates and cutoff determination. We will expand the sample size, particularly the positive panel, in follow-up studies to improve robustness. Fourth, given practical constraints, extensive independent batch repeats were not performed to fully quantify inter-run variability; thus, conclusions regarding assay robustness should be interpreted as preliminary. Fifth, cross-reactivity was not evaluated against major non-*Brucella* pathogens or serological confounders due to limited access to relevant strains; therefore, extrapolation of specificity to potentially cross-reactive field settings should be made with caution. Finally, multi-antigen formats were not exhaustively optimized (total coating amount, antigen ratios, mixed vs. sequential coating), and the underperformance of the four-antigen mixture under current conditions does not preclude potential improvement through systematic optimization. A pragmatic next step would be to use the tri-antigen panel as a baseline and refine the system along a ratio–density–background–cutoff framework with external validation (39).

## 6 Conclusion

This study established and optimized an iELISA using recombinant Brucella outer membrane–associated proteins (rOmp19, rOmp2b, rOmp31, and rBP26). Single-antigen assays showed inherent limitations, whereas multi-antigen combinations provided complementary diagnostic benefit. Within this dataset and under the current assay conditions, the tri-antigen panel (rOmp19 + rOmp31 + rBP26) showed a comparatively favorable sensitivity–specificity trade-off and is a reasonable candidate for further optimization. However, the present work should be viewed as antigen screening and preliminary evaluation rather than evidence for immediate high-precision serological diagnosis or field deployment. Given the lack of animal-level information on infection stage, the limited positive sample size (partly due to low prevalence in dispersed smallholder-source animals), and the lack of cross-reactivity testing, the evidence is insufficient to support immediate development or operational use of a new diagnostic strategy. Moreover, the antigen-dependent behavior observed in cELISA/SAT-discordant sera suggests that diagnostic ambiguity may persist in real-world settings and should be explicitly considered during validation. Future validation should include well-characterized controlled infection models with stage stratification, larger-scale field testing, and cross-reactivity assessment with stratified evaluation by health status/co-infections to confirm robustness in real-world settings.

## Data Availability

The datasets generated and/or analyzed during the current study are available in the NCBI GenBank (https://www.ncbi.nlm.nih.gov/) repository; GenBank accession *omp19* (https://www.ncbi.nlm.nih.gov/gene?term=ON813304.1), GenBank accession *omp2b* (https://www.ncbi.nlm.nih.gov/search/all/?term=AM712080.1), GenBank accession *omp31* (https://www.ncbi.nlm.nih.gov/gene/?term=MK611562.1) and GenBank accession *bp26* (https://www.ncbi.nlm.nih.gov/search/all/?term=AY166768.1).
