# Supplementary material for: Optimizing ovine brucellosis serodiagnosis: evaluation of recombinant Brucella antigens and multi-antigen combinations for iELISA
Source: Front Vet Sci. 2026 Mar 9;13:1775573. doi: 10.3389/fvets.2026.1775573 (PMC13006224; doi:10.3389/fvets.2026.1775573)
Supplement: Supplementary file 1 [file Data_Sheet_S1.DOCX]

**Table S1.** Densitometric assessment of recombinant protein purity from Coomassie-stained SDS–PAGE gels (two independent runs).

| **Sample** | **Main area (rep1)** | **Total area (rep1)** | **Purity (%) (rep1)** | **Main area (rep2)** | **Total area (rep2)** | **Purity (%) (rep2)** | **Mean purity (%)** | **SD purity (%)** |
| --- | --- | --- | --- | --- | --- | --- | --- | --- |
| rOmp19 | 2500 | 2575 | 97.09 | 2800 | 2856 | 98.04 | 97.56 | 0.67 |
| rOmp2b | 2331.08 | 2403.89 | 96.97 | 3260.02 | 3260.02 | 100 | 98.49 | 2.14 |
| rOmp31 | 2391.7 | 2391.7 | 100 | 2260.64 | 2260.64 | 100 | 100 | 0 |
| rBP26 | 2214.09 | 2214.09 | 100 | 2653.55 | 2653.55 | 100 | 100 | 0 |

**Note:** Band intensities were quantified by densitometry in ImageJ. “Main area” denotes the integrated density of the target protein band, and “Total area” denotes the summed integrated density of all detectable bands within the same lane after background subtraction. Purity (%) was calculated as (Main area / Total area) × 100 for each run (rep1 and rep2). Mean purity (%) and SD were calculated from the two independent runs.

**Table S2.** iELISA detection results of different antigens and their combinations in serologically discordant sera (cELISA and SAT).

| **Antigen** | **cELISA^+^SAT^-^**  **Positive (n=50)** | **cELISA^+^SAT^-^**  **Negative (n=50)** | **cELISA^-^SAT^+^**  **Positive (n=10)** | **cELISA^-^SAT^+^**  **Negative (n=10)** |
| --- | --- | --- | --- | --- |
| rOmp19 | 38/50 (76%) | 12/50 (24%) | 8/10 (80%) | 2/10 (20%) |
| rOmp2b | 20/50 (40%) | 30/50 (60%) | 8/10 (80%) | 2/10 (20%) |
| rOmp31 | 23/50 (46%) | 27/50 (54%) | 6/10 (60%) | 4/10 (40%) |
| rBP26 | 28/50 (56%) | 22/50 (44%) | 8/10 (80%) | 2/10 (20%) |
| rOmp19+rBP26 | 30/50 (60%) | 20/50 (40%) | 7/10 (70%) | 3/10 (30%) |
| rOmp19+rOmp31+rBP26 | 27/50 (54%) | 23/50 (46%) | 6/10 (60%) | 4/10 (40%) |
| rOmp19+rOmp2b+rOmp31+rBP26 | 36/50 (72%) | 14/50 (28%) | 8/10 (80%) | 2/10 (20%) |

**Note:** This table presents the detection results of 60 serologically discordant samples, including cELISA+SAT− samples (n=50) and cELISA−SAT+ samples (n=10). The samples were tested using the following antigen combinations: rOmp19, rOmp2b, rOmp31, rBP26, rOmp19 + rBP26, rOmp19 + rOmp31 + rBP26, and rOmp19 + rOmp2b + rOmp31 + rBP26. The results were classified as positive or negative based on the respective cutoff values for each antigen combination and are presented as percentages.


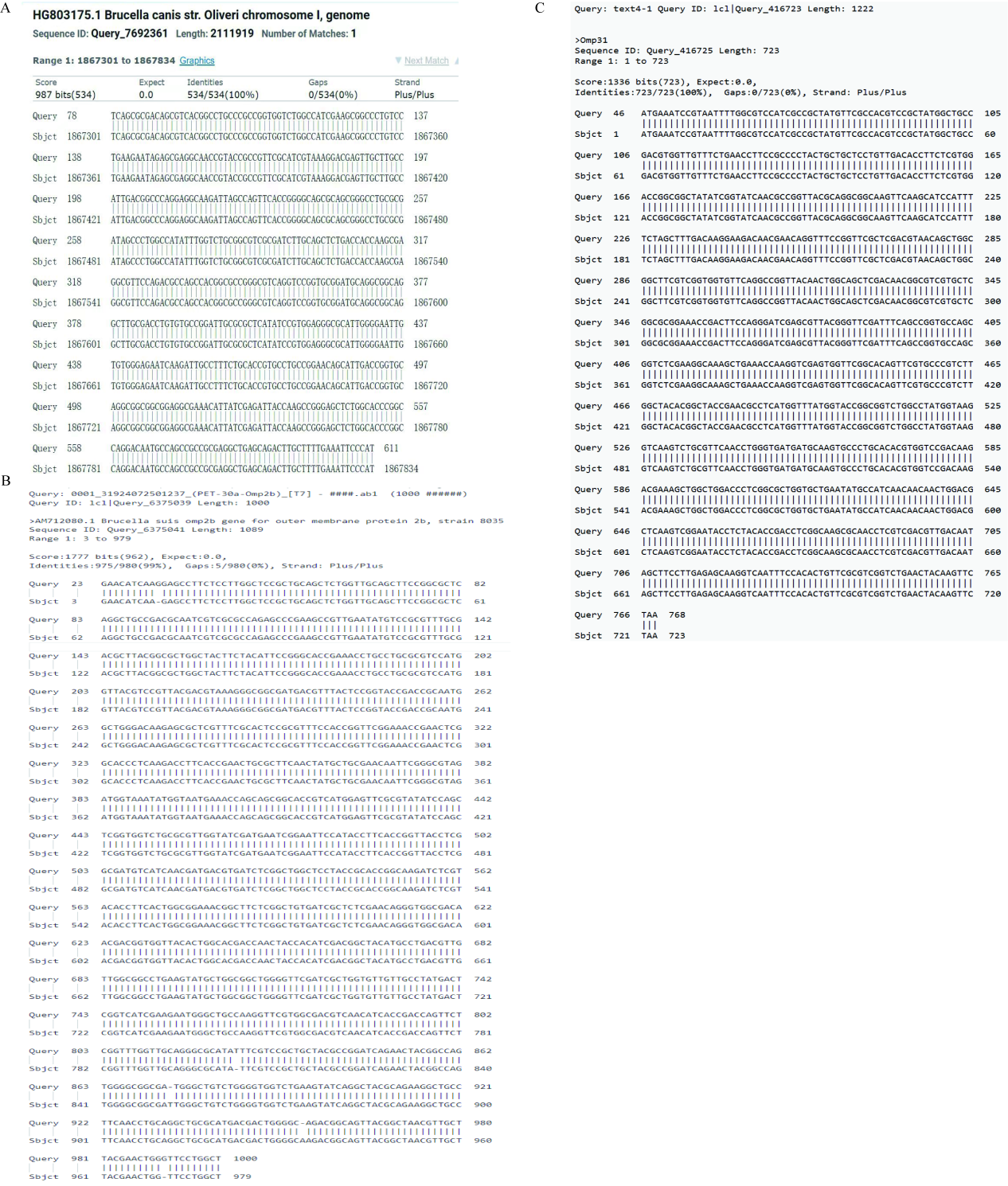


**Figure S1. NCBI BLAST alignment of Sanger sequencing reads from recombinant plasmids.**
Representative BLAST output showing alignment of Sanger sequencing reads from pET-30a/omp19 (A), pET-30a/omp2b (B), and pET-30a/omp31 (C) against reference *Brucella* gene sequences.


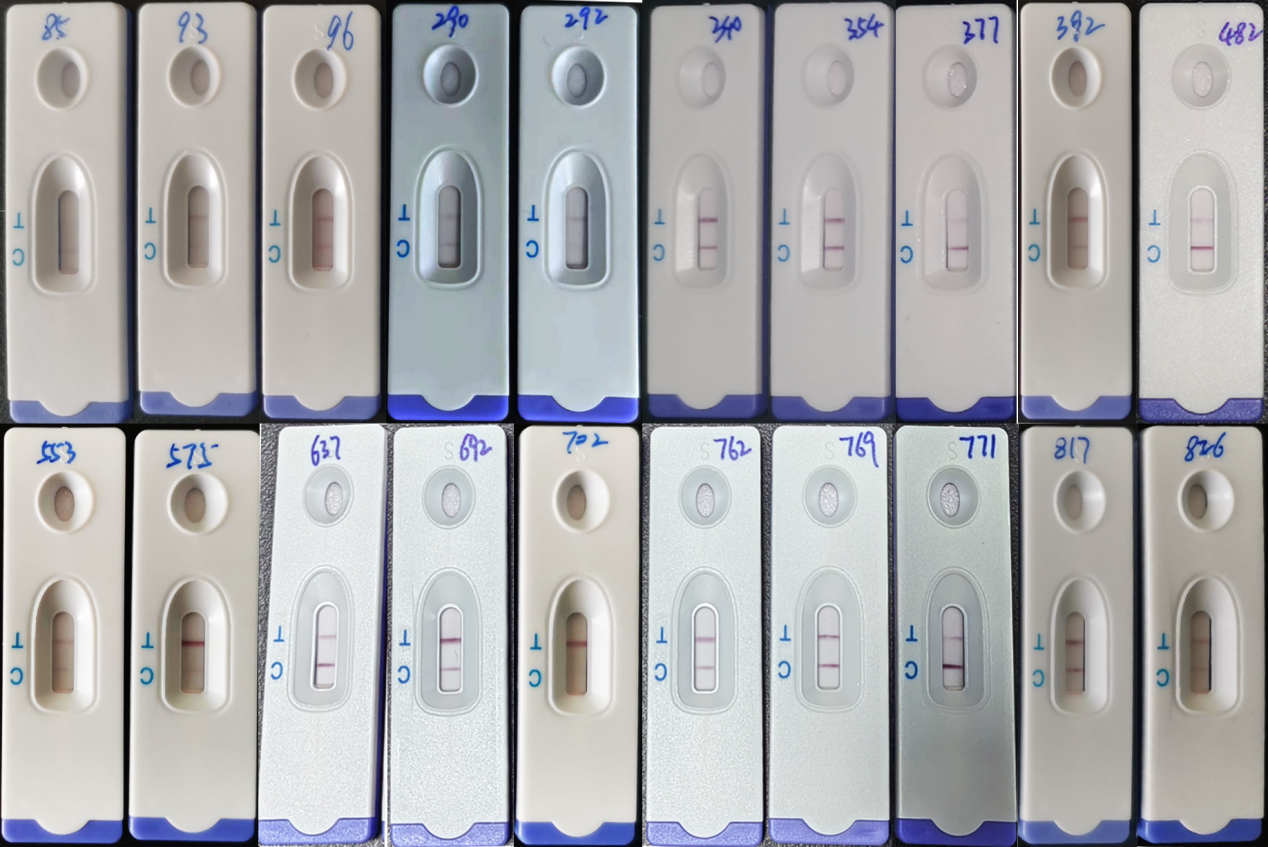
**Figure S2.** Colloidal gold LFIA confirmation of candidate seropositive sera.

Representative lateral flow immunoassay (LFIA) cassettes used to re-test sera classified as concordantly positive by cELISA and SAT (cELISA+/SAT+, n = 20).


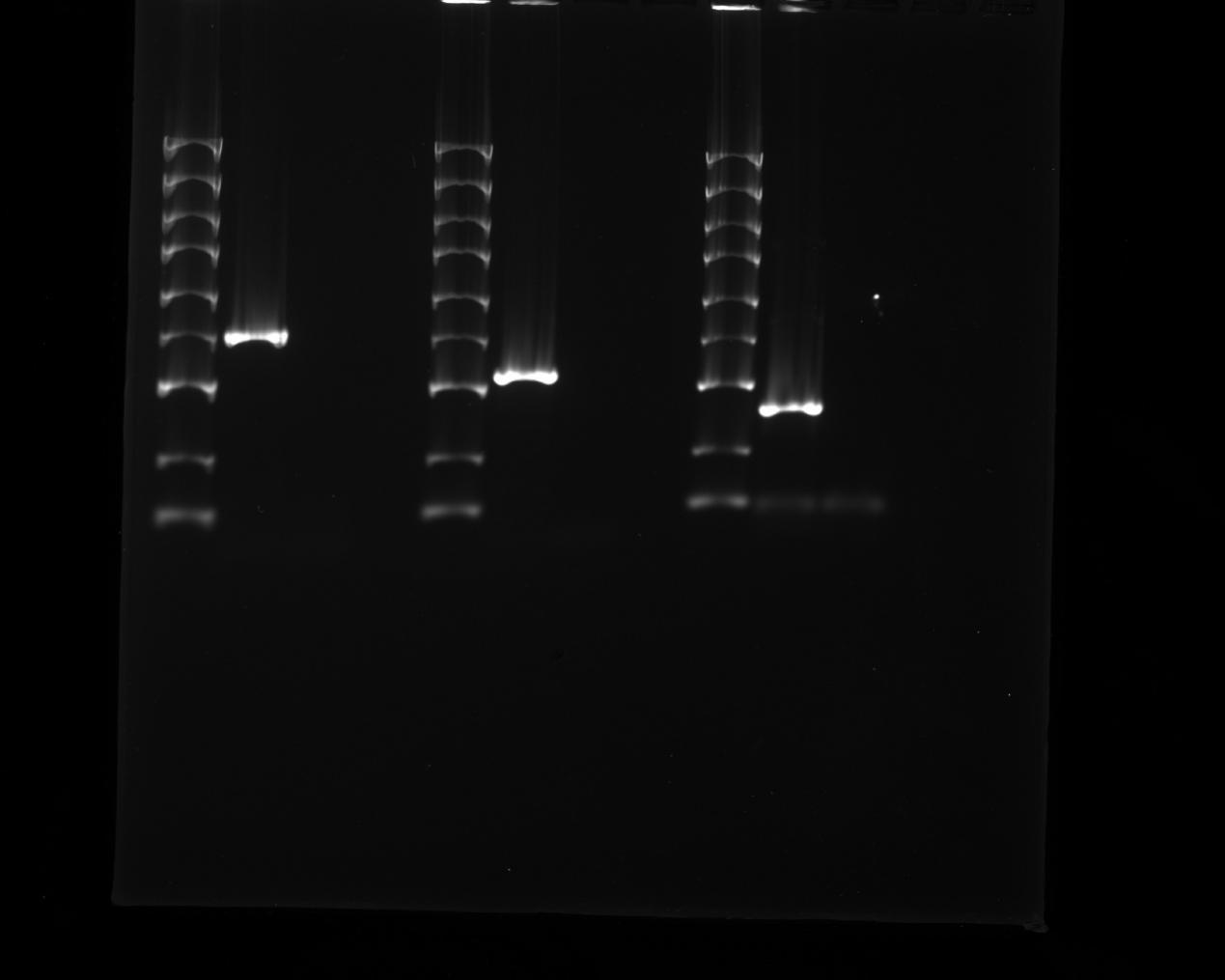


**Figure S3.** *omp31* and *omp19* PCR product amplification patterns.

Lane1, 3: DNA marker (100-5000bp); lane 2: *omp31*; lane 4: *omp19*


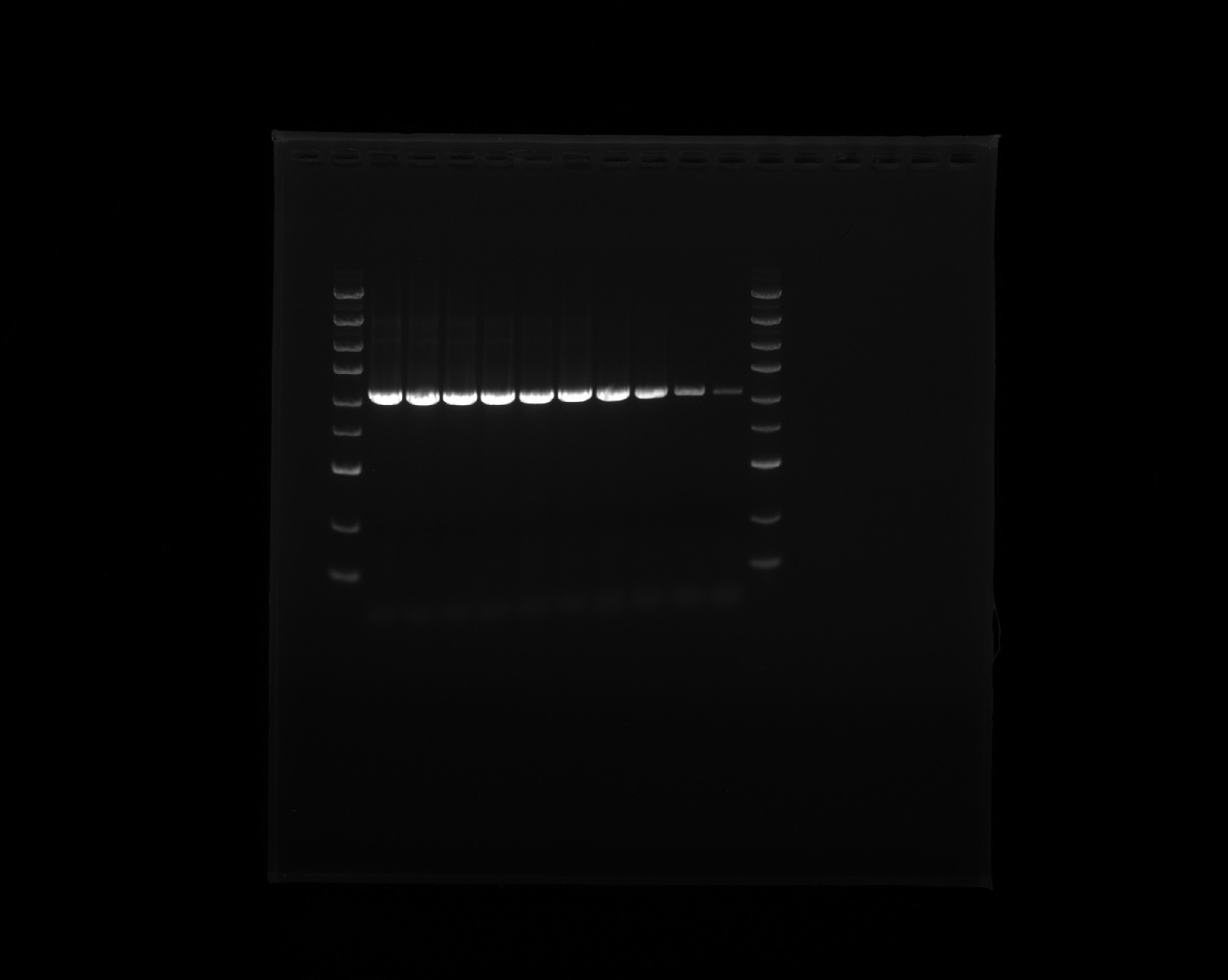


**Figure S4.** *omp2b* PCR product amplification pattern.

lane1,12: DNA marker (100-5000bp); lane2-11: *omp2b*.


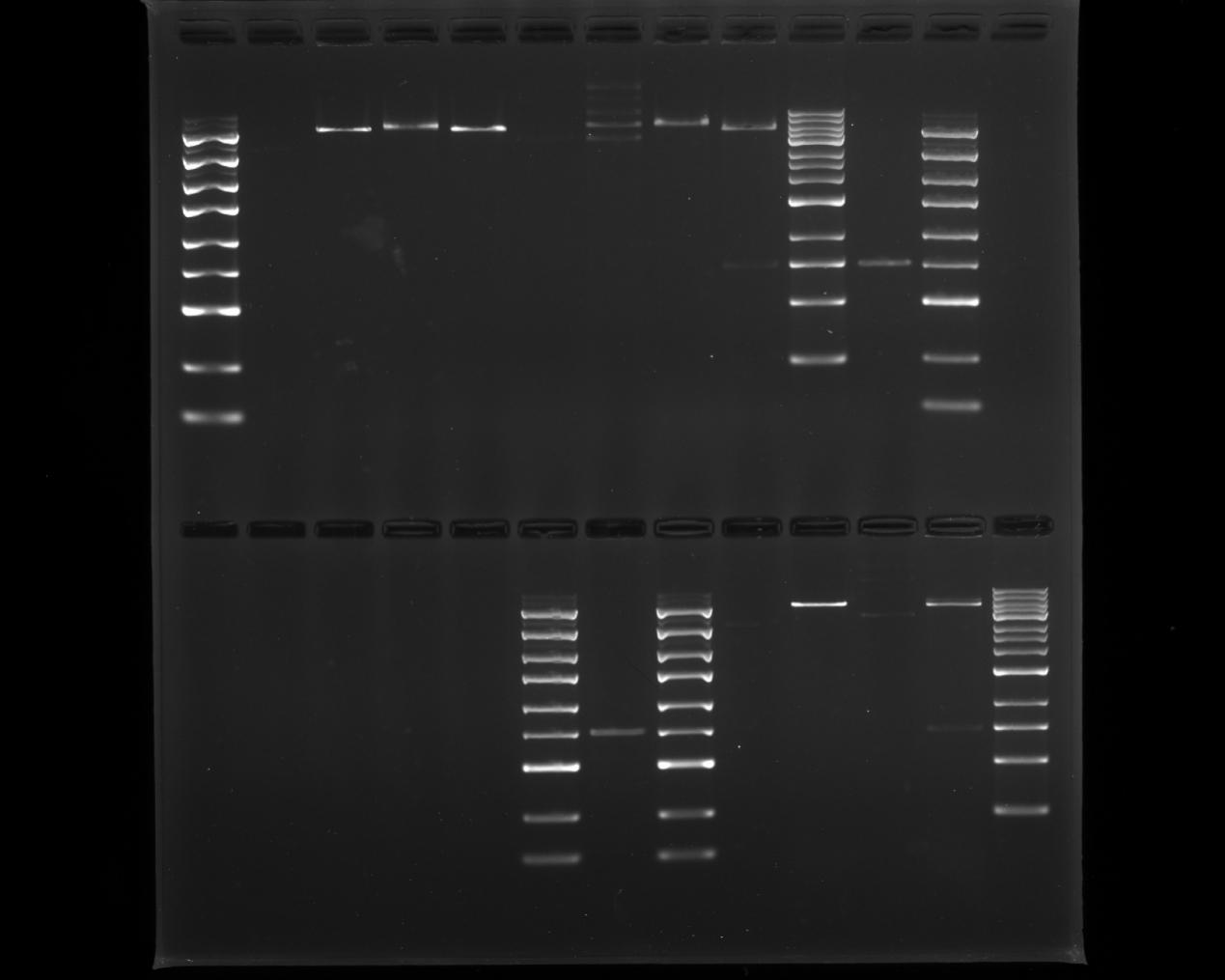


**Figure S5.** Agarose gel electrophoresis analysis of the recombinant plasmid pET-30a/omp31.

Lane 1 and lane 3: DNA marker (100–5000 bp); lane 2: *omp31* PCR product; lane 4: undigested recombinant plasmid pET-30a/omp31; lane 5: recombinant plasmid pET-30a/omp31 digested with EcoRI; lane 6: recombinant plasmid pET-30a/omp31 digested with HindIII; lane 7: recombinant plasmid pET-30a/omp31 digested with EcoRI and HindIII; lane 8: DNA marker (250–12,000 bp).


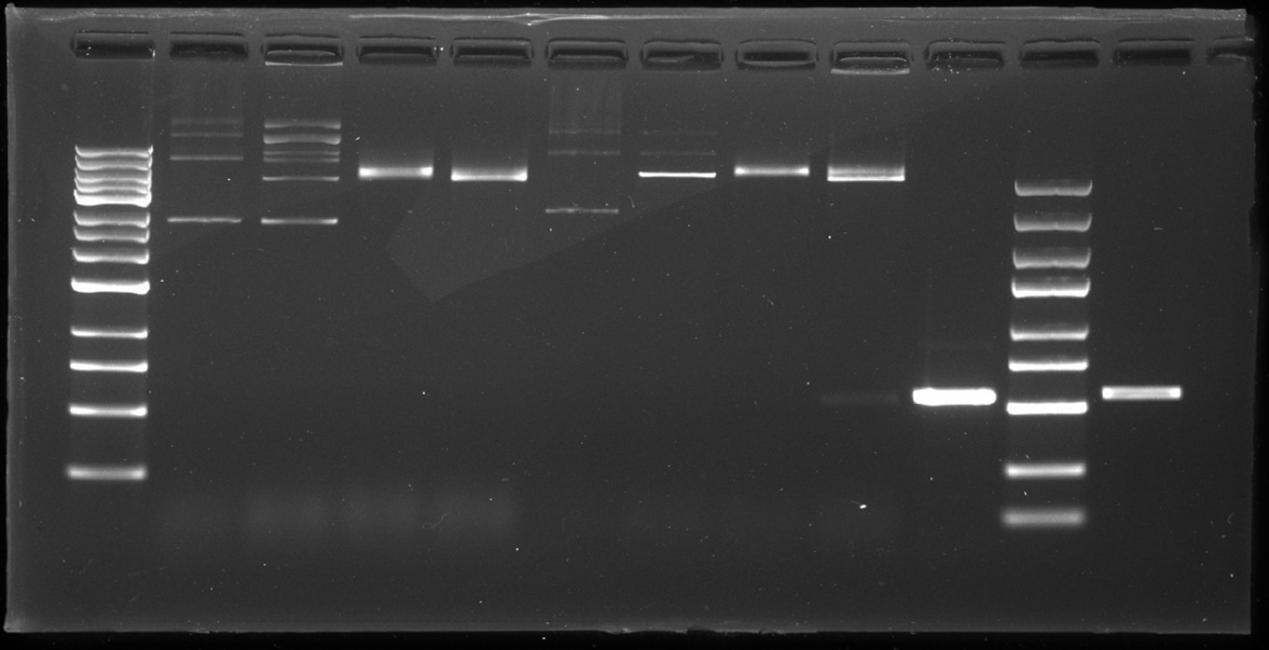


**Figure S6.** Agarose gel electrophoresis analysis of the recombinant plasmid pET-30a/omp19.
Lane 1: DNA marker (250–12,000 bp); lane 2: undigested pET-30a vector plasmid; lane 3: pET-30a vector plasmid digested with EcoRI; lane 4: pET-30a vector plasmid digested with HindIII; lane 5: pET-30a vector plasmid digested with EcoRI and HindIII; lane 6: undigested recombinant plasmid pET-30a/omp19; lane 7: recombinant plasmid pET-30a/omp19 digested with EcoRI; lane 8: recombinant plasmid pET-30a/omp19 digested with HindIII; lane 9: recombinant plasmid pET-30a/omp19 digested with EcoRI and HindIII; lane 10: *omp19* PCR product; lane 11: DNA marker (10–5,000 bp).


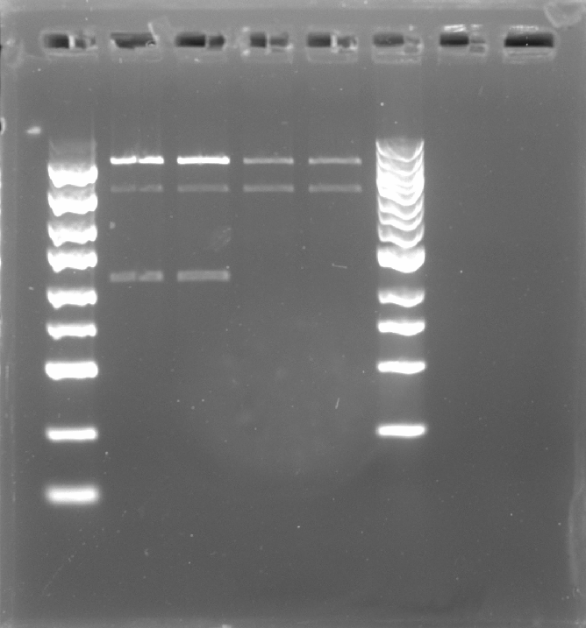


**Figure S7.** Agarose gel electrophoresis analysis of the recombinant plasmid pET-30a/omp2b.
Lane 1: DNA marker (10–5,000 bp); lanes 2–3: recombinant plasmid pET-30a/omp2b digested with EcoRI and HindIII; lanes 4–5: undigested recombinant plasmid pET-30a/omp2b; lane 6: DNA marker (250–12,000bp).


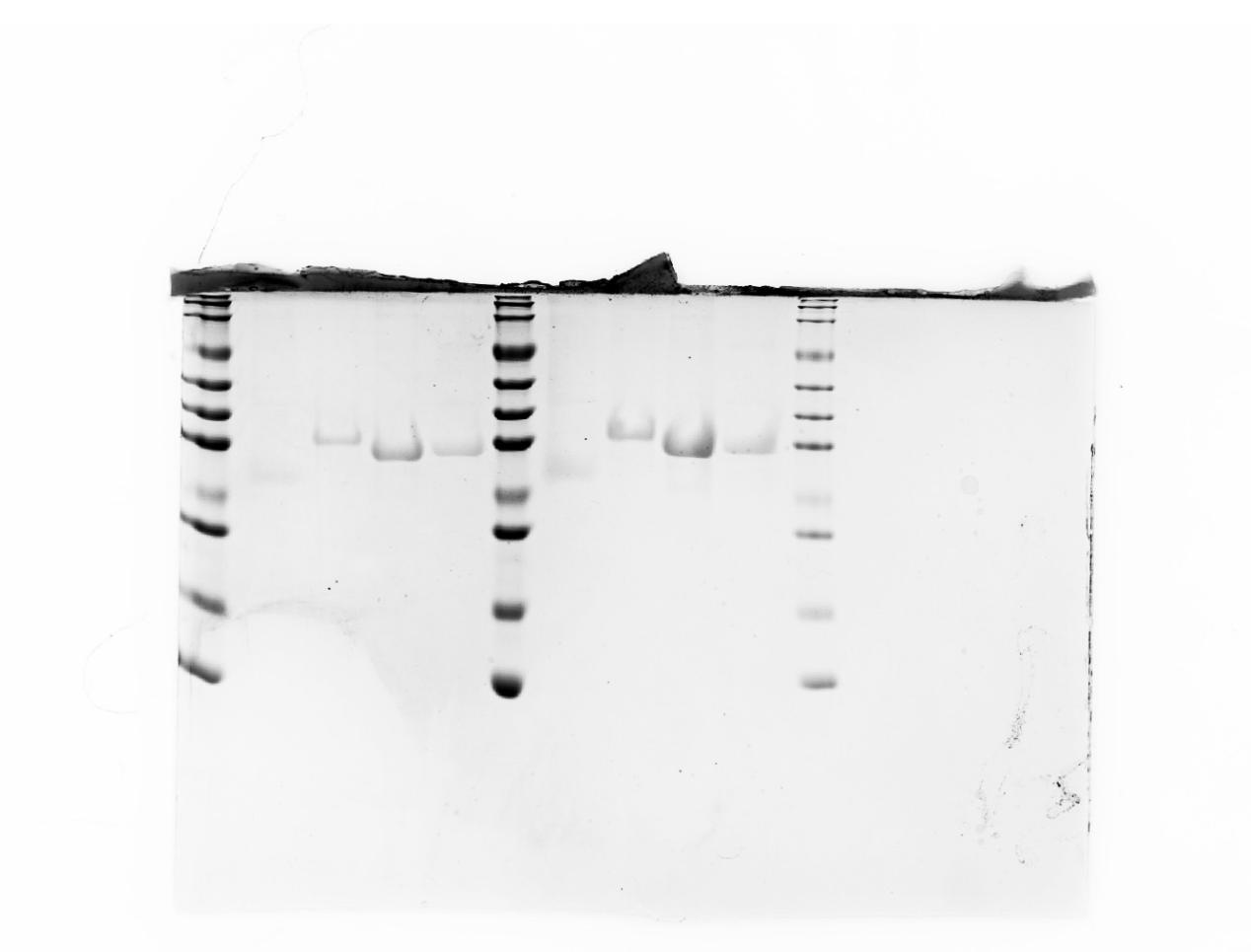


**Figure S8.** SDS–PAGE analysis of purified recombinant proteins.
Lane 1 and lane 6: protein marker (10–250 kDa); lane 2: purified rOmp19; lane 3: purified rOmp2b; lane 4: purified rOmp31; lane 5: purified rBP26.


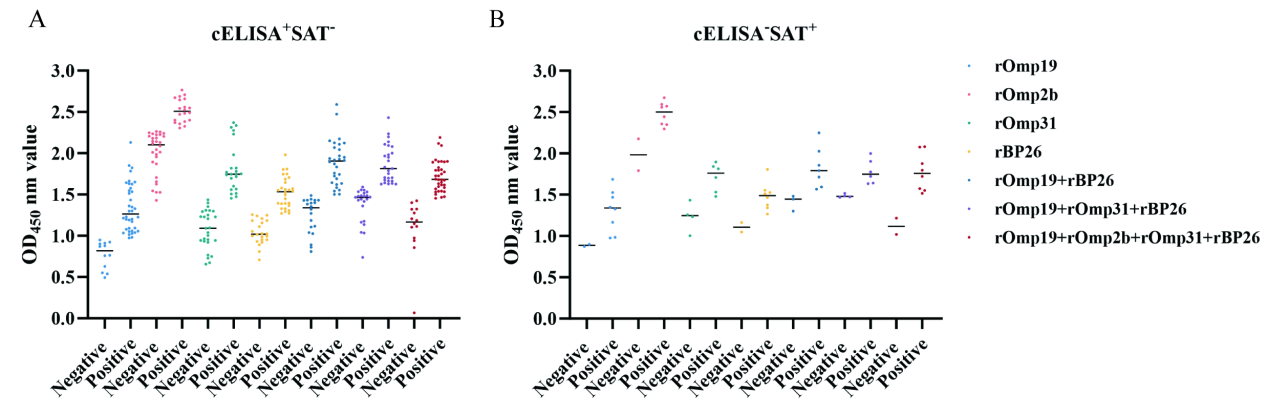


**Figure S9.** iELISA OD_450_ value distribution in serologically discordant sera from cELISA and SAT. This figure shows the OD_450_ value distribution of 60 serologically discordant samples, including 50 cELISA+SAT− samples (A) and 10 cELISA−SAT+ samples (B). The samples were tested using rOmp19, rOmp2b, rOmp31, rBP26, rOmp19 + rBP26, rOmp19 + rOmp31 + rBP26, and rOmp19 + rOmp2b + rOmp31 + rBP26 as antigens, under optimal conditions for iELISA. The x-axis represents the different antigen combinations, with samples classified as positive or negative based on their respective cutoff values. The y-axis indicates the OD_450_ values, with each data point representing one sample.
